# Non-surgical root canal treatments in a public dental service: Characteristics of patients, teeth and operators from a 16-year register-based study

**DOI:** 10.1007/s00784-026-06876-x

**Published:** 2026-04-24

**Authors:** Milo Väisänen, Ulla Palotie, Jussi Furuholm, Battsetseg Tseveenjav

**Affiliations:** 1 Faculty of Medicine, Department of Oral and Maxillofacial Diseases, University of Helsinki, Helsinki, Finland; 2 Department of Oral and Maxillofacial Diseases, University of Helsinki, Helsinki University Hospital, Helsinki, Finland; 3 Institute of Dentistry, University of Turku, Turku, Finland; 4 Department of Maxillofacial Surgery, Päijät-Häme Joint Authority for Health and Wellbeing, Päijät-Häme Central Hospital, Lahti, Finland

**Keywords:** Root canal treatment, Endodontics, Retrospective studies, Dental pulp diseases, Epidemiology

## Abstract

**Objectives:**

According to Finnish population-based studies, 27% of dentate adults have at least one tooth with apical periodontitis (AP), and 61% have at least one root canal treated teeth (RCTT). The objective of this retrospective register-based study was to assess the prevalence of non-surgical root canal treatments (nsRCTs) performed at public dental service (PDS) of the City of Helsinki, and to describe the patient-, tooth- and operator-related factors of these treatments.

**Material and methods:**

Data were extracted from electonic patient files used by Helsinki PDS between 2002 and 2017. NsRCTs were identified based on dental treatment codes on nsRCT initiation and root canal filling.

**Results:**

A total of 166 218 teeth were identified with a treatment code for nsRCT. Of these, 81.4% was completed; 18.6% uncompleted nsRCTs. Younger adults and women more frequently received nsRCTs, compared with older counterparts and men (*p* < 0.001). At tooth level, molars received more nsRCTs than anterior teeth or premolars (*p* < 0.001). Most of the nsRCTs were initiated as planned treatment compared to those initiated at emergency visits, and performed by general dentists (GDs).

**Conclusion:**

A substantial number of nsRCTs were performed annually at Helsinki PDS. The proportion of uncompleted nsRCTs was high, indicating inefficient use of resources. Our findings of patient-, tooth- and operator-related characteristics could provide new insights into the epidemiology of endodontics in a public dental care service.

**Clinical relevance:**

While nsRCT is a common procedure in dentistry, findings from 16-year register-based study could support clinicians and decision-makers for their evidence-based treatment and management.

## Introduction

The oral health of Finnish adults has improved considerably over the past 30 years across the entire Finnish population, with the most prominent change being reduced edentulousness [1–3]. As more and more adults retain their natural teeth throughout life, the demand for non-surgical root canal treatments (nsRCTs) is expected to increase. According to the nationwide Health 2000 study, 27% of Finnish adults have apical periodontitis (AP) [1]. Further, 61% of Finnish dentate adults aged ≥ 30 years have radiographic evidence of at least one root canal treated tooth (RCTT), with predominance among older adults and women [4]. This is higher than corresponding rates for Danish [5] and Norwegian [6] adults. At tooth level, among Finnish adults included in Health 2000 study [4], 7% of of all teeth were root canal treated, while the corresponding proportion in Swedish study was 6.3% [7]. Worldwide, over half of adults have at least one RCTT [8].

The Finnish public dental service (PDS) began providing subsidized services to younger age groups of adults since the 1980 s, and all remaining age restrictions were removed in 2002 [9]. After the reform, the annual attendance of Finnish adults in PDS has been found to be 31.9% [10]. In the city of Helsinki, a recent publication showed that the overall annual attendance of adults aged ≥ 20 years in public and private dental care together was between 48.8 and 51.9% from the period 2007–2017 [11]. Of all treatments provided in the Finnish PDS, endodontic treatments accounted for 3.8% in 2009 [12] and 5.3% from 2001 to 2013 [13].

Characteristics of individuals and teeth receiving nsRCTs as well as outcomes of these treatments have been investigated in other register-based studies from Scandinavia [14–17]. In Sweden, average age of individuals at the time of root filling was 55 years (range 20–102), and maxillary teeth and mandibular first molars were most frequently root-filled [17]. Similar results were found in another Swedish study from PDS where molars most often received nsRCTs, although the mean age was lower 48.3 (SD = 16.4) [18]. With respect to operators, it is known that nsRCTs are carried out by both general dentists (GDs) and endodontists. Differences exist between operators when comparing treatment decisions [19] or quality of root fillings [20]. NsRCTs are also considered technically challenging and stressful by GDs, particularly regarding on molars [21]. Consequently, referrals of GDs to endodontists are more common in molar teeth [22], and molars are usually more often treated by endodontists in some countries [23].

This study examined the background of individuals who received nsRCTs at Helsinki PDS, and the distribution of these treatments by patient-, tooth- and operator-related factors. In Finland, the prevalence of nsRCT at PDS has not been studied in the entire population-level. There is a need for such study with long-term observation years to analyze and learn from our past.

### Objective

The aim of this study was to assess the prevalence of nsRCTs performed on adult patients at Helsinki PDS based on a 16-year observation period between 2002 and 2017. In addition, this study also sought to investigate patient-, tooth- and operator-related characteristics of nsRCTs, carried out during the observation period.

### Materials and methods

Data were collected from two electronic patient file systems EFFICA and Lifecare (Tieto Oyj, Helsinki, Finland) which were used by Helsinki PDS clinics as well as by purchased service GDs between 2002 and 2017. Data were extracted from the patient files based on dental treatment codes maintained by the National Institute for Health and Welfare (THL). Explanation of the treatment codes can be found in Appendix A. NsRCTs at Helsinki PDS are provided by public GDs, endodontists and dental students. Also, the PDS purchased GD services to manage and decrease waiting times. This service included nsRCTs among other dental treatments.

The inclusion criteria were adult patients aged ≥ 18 years with a nsRCT treatment code in their dental history for any permanent teeth. Procedures on third molars or primary teeth were excluded. Teeth were numbered according to the ISO 3950 system, where the teeth are charted with quadrant number (1-maxillary right, 2-maxillary left, 3-mandibular left, 4-mandibular right), followed by the tooth number, which starts from the midline of each jaw from 1 to 7. Completed nsRCTs were treatments where treatment code on root canal filling was found in contrast to uncompleted nsRCTs where only treatment code for nsRCT initiation was found. Primary nsRCT was defined as the first completed treatment found in the patient files. Retreatment denoted a further completed nsRCT found for the same tooth. No radiographs or clinical parameters were evaluated in this study.

Patient-related characteristics were age and sex. Tooth-related information was tooth type and jaw. Patient age was grouped into the following five categories: 18–29, 30–49, 50–64, 65–74 and 75 + years. The nature of dental visit for nsRCT initiation was inferable from dental treatment codes. Operators who performed nsRCTs were grouped into GDs in public or purchased service, endodontists and dental students. Operator-related information was available from the years 2002–2016. Therefore, analyses with operator-related information included lower number of cases than those until 2017.

### Ethical considerations

Data were acquired from encrypted electronic patient file systems. No individuals were identified and ethical permission was not required. Permission for this study was granted by the City of Helsinki (Research permission HEL2022-013077).

### Statistical analyses

Further data processing was performed with IBM SPSS Statistics (Version 31.0.0.0). Difference in mean ages between groups was tested with Student’s test with assumption of equal variances. χ^2^ test was used to investigate differences between categorical variables. Post-hoc testing was made with Bonferroni correction. Pearson correlation was used to test statistical significance in the trendline of annual completed nsRCTs. Due to large sample size, p-values ≤ 0.001 were considered as statistically significant.

## Results

### Completed and uncompleted non-surgical root canal treatments

**Fig. 1:**
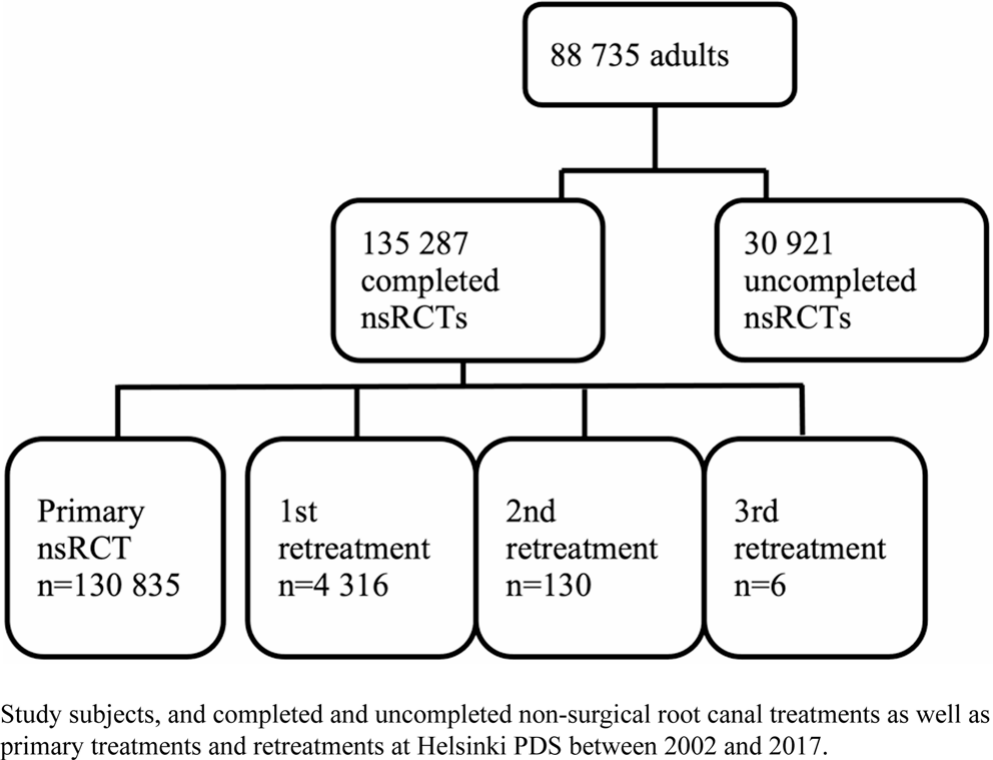
Completed and uncompleted non-surgical root canal treatments

According to electronic patient files, 166 208 teeth with treatment code related to nsRCT were found at Helsinki PDS during the 16-year observation period. Of these, 18.6% (*n* = 30 921) were uncompleted as they had only nsRCT initiation without root canal filling. Completed primary nsRCTs accounted for 78.7% of all cases, and the rest were retreatments (Fig. 1). Completed nsRCTs belonged in total to 88 735 different adults.

Women represented 52.8% of the completed nsRCT; men accounted for the remaining 47.2% of the cases. Similar distribution was found for uncompleted nsRCTs. In the group of completed nsRCTs, mean age at the time of nsRCT initiation was 43.1 (SD 15.1) years which was higher than for patients with uncompleted nsRCT (40.9 (SD 15.3)) (*p* < 0.001). For completed nsRCTs, molar teeth were the most commonly treated teeth (49.4%), followed by premolars (32.4%) and anterior teeth (18.2%). The overrepresantation of molars was even more evident for uncompleted nsRCTs (56.3%, *p* < 0.001). Finally, 31.4% of uncompleted nsRCTs were initiated as emergency treatments compared with approximately 9% for completed nsRCTs (*p* < 0.001).

### Prevalence of non-surgical root canal treatments by operators

**Fig. 2:**
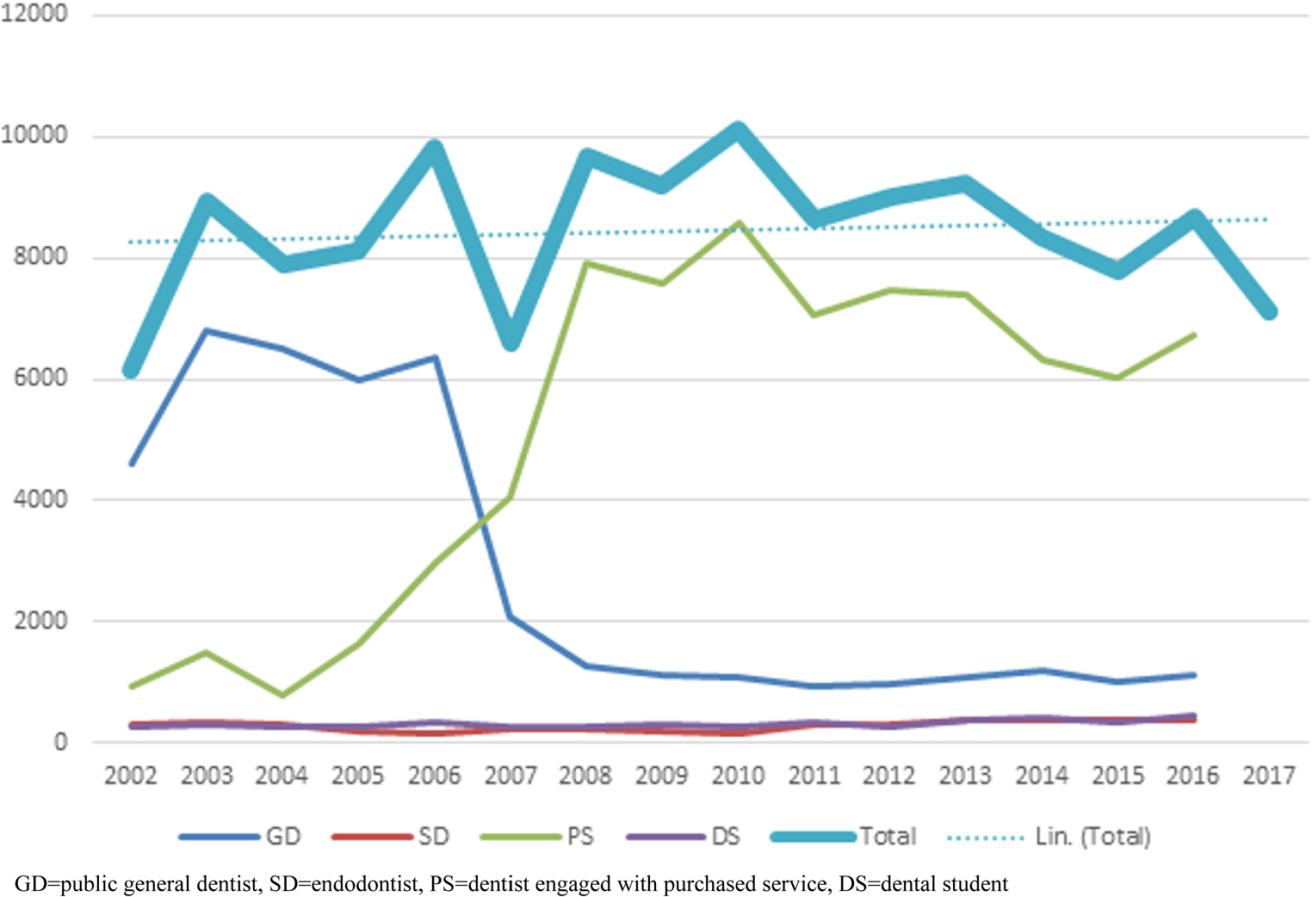
Prevalence of completed non-surgical root canal treatments at Helsinki Public Dental Service

The prevalence of completed nsRCTs ranged from 6 140 (in 2002) to 10 108 (in 2010). Most of the nsRCTs were performed by GDs; until 2006 by those engaged at PDS, and after by those in purchased service. Of all nsRCTs between 2002 and 2016, 60.0% (*n* = 76 939) were performed by GDs in purchased service, and 32.9% (*n* = 42 155) by public GDs (Table 1). Only 3.3% (*n* = 4 245) of nsRCTs were performed by endodontists and 3.8% by dental students (*n* = 4 844). No difference in the distribution of operators was observed when comparing with uncompleted nsRCTs. Trendline analysis revealed that the prevalence of completed nsRCTs did not differ by years (*p* = 0.867). The prevalence of completed nsRCTs by year of observation and operators is shown in Fig. 2.

**Table 1:** Distribution of completed non-surgical root canal treatments by tooth- and operator-related factors and nature of dental visit

Age	18–29	30–49	50–64	65–74	75–	All	*P*-value
	*n* (%)	*n* (%)	*n* (%)	*n* (%)	*n* (%)	(%)	
Tooth type							**< 0.001**
Anterior teeth	3683^a^	7943^a^	7153^b^	3503^c^	2313^d^	24,595	
	(12.6)	(12.6)	(24.5)	(36.6)	(51.8)	(18.2)	
Premolars	8587^a^	20,247^b^	10,169^c^	3456^c^	1395^a, b^	43,854	
	(29.4)	(32.2)	(34.8)	(36.1)	(31.2)	(32.4)	
Molars	16,912^a^ (58.0)	34,658^b^ (55.1)	11,886^c^ (40.7)	2624^d^ (27.4)	758^e^ (17.0)	66,838 (49.4)	
Tooth location							**< 0.001**
Maxilla	16,696^a^	35,589^a, b^	16,269^b^	5358^a, b^	2296^c^	76,208	
	(57.2)	(56.6)	(55.7)	(55.9)	(51.4)	(56.3)	
Mandible	12,486^a^	27,259^a, b^	12,939^b^	4225^a, b^	2170^c^	59,079	
	(42.8)	(43.4)	(44.3)	(44.1)	(48.6)	(43.7)	
Nature of dental visit							**< 0.001**
Planned	26,375^a^	56,772^b^	26,534^c^	8856^d^	4090^d^	122,627	
	(89.4)	(90.4)	(91.4)	(92.7)	(92.7)	(90.6)	
Emergency	3133^a^	5998^b^	2511^c^	694^d^	324^d^	12,660	
	(10.6)	(9.6)	(8.6)	(7.3)	(7.2)	(9.4)	
Operator							**< 0.001**
General dentist in purchased service	17,068^a^	36,556^a^	16,711^a^	5062^b^	1542^c^	76,939	
	(61.7)	(61.0)	(60.8)	(56.5)	(37.0)	(60.0)	
Public general dentist	9071^a^	19,835^a^	8234^b^	2782^b^	2233^c^	42,155	
	(32.8)	(33.1)	(30.0)	(31.1)	(53.6)	(32.9)	
Endodontist	825^a^	1787^a^	1020^b^	436^c^	177^b, c^	4245	
	(3.0)	(3.0)	(3.7)	(4.9)	(4.3)	(3.3)	
Dental student	693^a^	1743^b^	1523^c^	673^d^	212^c^	4844	
	(2.5)	(2.9)	(5.5)	(7.5)	(5.1)	(3.8)	
All	29,182	62,848	29,208	9583	4466	135,287	
	(46.2)	(46.5)	(21.6)	(7.1)	(3.3)	(100)	

Groups in the same row that do not share a common letter (a,b,c,d,e) differ statistically significantly (Bonferroni correction)

### Completed non-surgical root canal treatments by age groups

Of all nsRCTs, 89.6% were performed on those aged less than 65 years. There was a statistically significant difference between age groups regarding sex, tooth type, the nature of dental visit, and operators (Table 1). Among age groups ≥ 65 years, anterior teeth were more often treated than posterior teeth compared with younger age groups (*p* < 0.001). In contrast, molar teeth were more often treated in age groups of 18–29 years and 30–49 years and less often in age groups of 65–74 years and 75 ≥ years (*p* < 0.001). 

### Completed non-surgical root canal treatments by tooth type

**Fig. 3:**
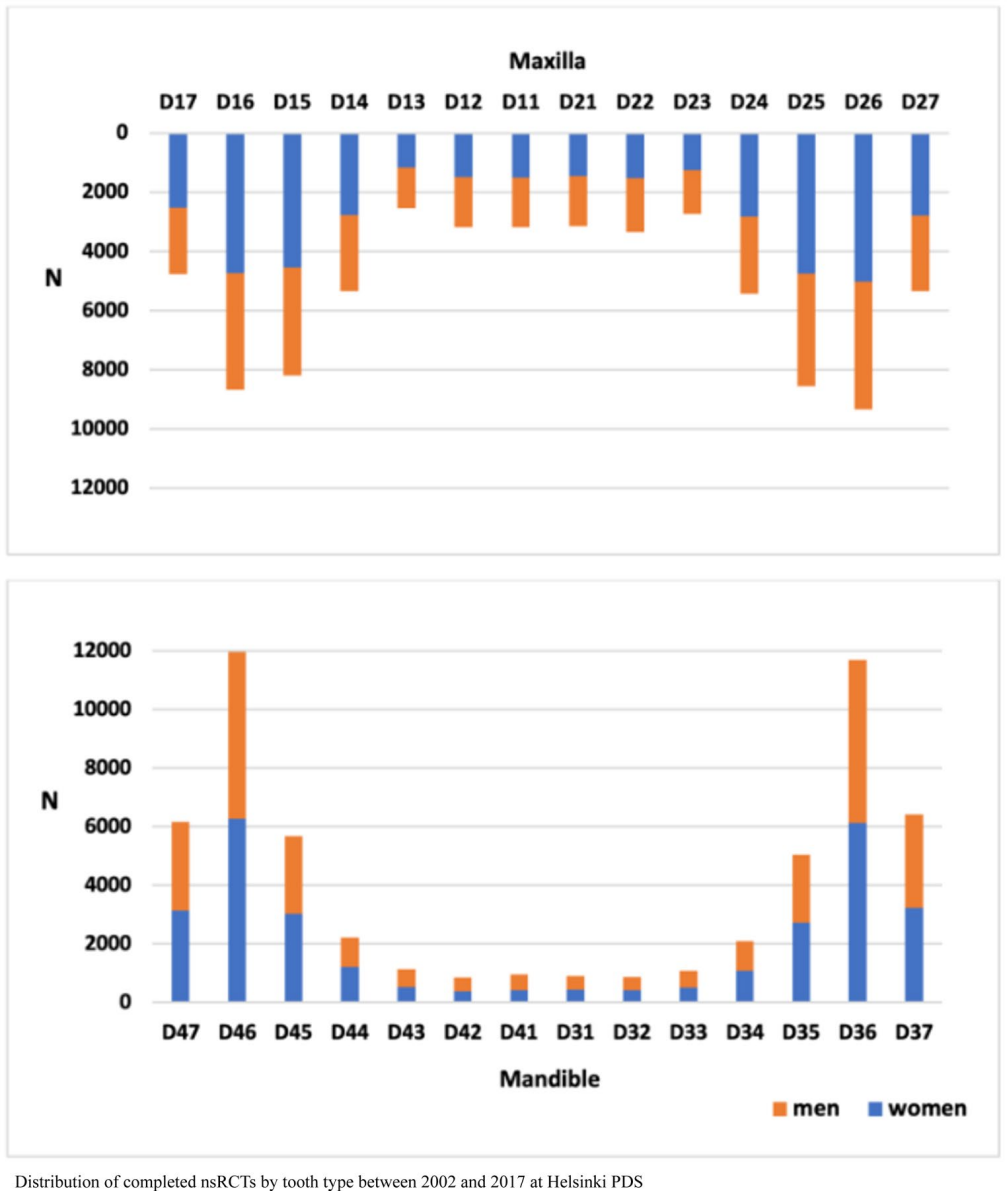
Prevalence of completed non-surgical root canal treatments by tooth type

In both maxilla and mandible, first molars were the most common teeth to receive nsRCTs (Fig. 3). First upper molars accounted for 24.8% of all the upper teeth, followed by second premolars (22.7%) and second molars (13.5%). First lower molars received 41.8% of nsRCTs of all lower teeth. In addition, lower second molars (21.9%) and second premolars (18.7%) frequently had nsRCTs. Tooth 46 received the most nsRCTs of the whole dentition, representing 9.2% of all teeth. Comparing nsRCTs by tooth type and operator, we found that molars were more often treated than anterior teeth by both endodontists and by public GDs, GDs in purchased service and dental students combined (*p* < 0.001). Molar teeth represented approximately 50% of all treated teeth in both groups (Table 2).

**Table 2:** Completed non-surgical root canal treatments by tooth type and operators

Tooth type	Anterior	Premolars	Molars	All	*P*-value
	*n* (%)	*n* (%)	*n* (%)		
Operators					
Endodontist	1296^a^ (30.5%)	1106^b^ (26.1%)	1843^b^ (43.4%)	4245	
Others	21,845^a^ (17.6%)	40,388^b^ (32.6%)	61,705^b^ (49.8%)	123,938	
All	23,141	41,494	63,548	128,183	< 0.001

Groups in the same row that do not share a common letter (^a,b^) differ statistically significantly (Bonferroni correction)

## Discussion

A significant number of nsRCTs were performed annually at Helsinki PDS during our study period. The prevalence of nsRCTs increased slightly at Helsinki PDS between 2002 and 2017 even though the trendline analysis was not statistically significant. A notable increase in the prevalence of nsRCTs seen in 2003 can be explained by the Finnish Dental Care Reform (2001–2002), which removed all age restrictions for utilization of public dental care [9]. It is worth exploring the amount of uncompleted nsRCTs found in this study as they accounted for 18.6% of all nsRCTs. This could be partly explained by the fact that patients have had continuation in other dental care provider, such as private sector or moved to another municipality. It is possible that dental fear played a role in patients not attending subsequent appointment to complete the nsRCT once their pain had ceased [24]. This is supported by our findings that almost one third of uncompleted nsRCTs were initiated as emergency treatment of which the main purpose is to eliminate pain. Also, extractions could have been performed after nsRCT initiation explaining loss of cases. Nevertheless, it is not unusual that nsRCTs are not completed [25]. Consequently, enhanced communication between dentists and patients is required to discuss the importance of completion of endodontic treatment. Uncompleted nsRCTs could present a threat for patients health as there is an increased risk for spread of infection and hospitalisation [26]. Further studies are needed to examine the reasons for uncompleted nsRCTs, as this represents also a challenge for improving the efficiency of the PDS.

Dental services in Finland are typically provided by either public or private organizations. The system has gone through multiple reforms, of which that in 2001–2002 is the most important during our study period [9]. Inequality and inequity still remain major problems in the use of dental services [27]. Nevertheless, the number of adults visiting PDS and treatment measures have been increasing during our study period, at least in southern Finland [13]. Similarly, endodontic treatments have had a statistically significant increasing trend among those aged 18–39 years. However, a declining trend in endodontic procedure codes has been reported in both public and private dental care over an 11-year period in the city of Helsinki, although their use of aggregated data limits the comparability with our findings [11]. Additionally, the prevalence of RCTT has decreased in the Finnish private sector, from 83 241 in 2012 to 61 188 in 2017 [28]. Further research is required to confirm and better understand this observed trend.

Although our data demonstrated an increasing trend in the prevalence of nsRCTs, it is challenging to make further conclusions. Differences between studies in the prevalence of RCTT or nsRCT may be due to study methods and population. One may hypothesize that tooth extraction and replacement by implant has become more frequent. In a large nationwide observational study from Finland, the mean number of extractions per patient was 0.17 in 2012 and 0.18 in 2017 in private sector, which does not support the hypothesis of fewer extractions [29]. Another study found that at Helsinki PDS, the rate of extractions increased as a function of age, and rate of endodontic treatments decreased even though the trend in extractions did not change over time [30]. However, only adults aged ≥ 60 years were investigated.

We found that age group of 30–49 years received most of the completed nsRCTs. In private Finnish dental clinics, the weighted mean age of an endodontic patient was 53.6 years in 2012 and 55.9 years in 2017 [28]. In our study, mean age at the time of nsRCT initiation was considerably lower being 43.1 (SD 15.1). This is also lower than in one comparable Swedish study [18] but similar to a study conducted at PDS in Northern Finland [31]. This suggests that patients at Finnish PDS are usually younger than in private sector [11]. Additionally, we found that the prevalence of nsRCTs decreased continually in every age category without any peaks in the oldest groups, which has been observed in other studies [32, 33]. Between age groups, we found statistical differences by sex, tooth type, nature of dental visit and operators. The greatest difference in relative proportions of nsRCTs was found by tooth type. Molar teeth were less often treated among elderly patients at Helsinki PDS as they received proportionally more nsRCTs in anterior teeth. For instance, in the age group of 75 + years, approximately half of all nsRCTs were performed in anterior teeth, compared with 12.6% for those aged 18–29 years. As elderly adults have usually less teeth than younger adults and molars are more often extracted in older adults [34], our findings are not unusual.

A large majority of the completed nsRCTs performed were planned in every age category. The proportion of emergency treatments was approximately 10% of all the nsRCTs for most of the age categories. Some of the adults with an assessed need for nsRCT that started as emergency care were referred to purchase service of the city for planned treatments to be completed by GDs. This practice of combining public and purchased service was due to disequilibrium of demand of oral health treatment and supplier capacity at PDS. As a register-based study, we do not know the reasons for emergency treatment. It is possible that they included trauma or acute pain due to inflammation of the pulp or periradicular conditions [35]. It should be noted that the use of treatment codes in emergency appointments might have not been consistent within dentists, and it is possible that treatment codes other than those we used for calculations could have been used.

We found that both in maxilla and in mandible, posterior teeth more often received nsRCTs than the anterior teeth being consistent with a study from Swedish PDS [18]. First lower molars were the most often treated teeth aligning with a previous study [17]. One cross-sectional study of over 20 000 teeth found that AP was most prevalent in the first molars [36]. Hence, molars may be more affected by caries [37], and thus by pulpitis and AP, while anterior teeth may require nsRCTs more often due to trauma. Difficulties of maintaining proper oral health in the posterior region of the mouth and a larger tooth surface may favour decay development in molars compared with anterior teeth. Additionally, early eruption of molars may cause accumulation of treatments over a long time period on these teeth. Finally, we compared whether tooth groups differed by operators. We found that all operators performed nsRCTs more on molars than on anterior teeth which is the opposite of the pattern reported for Finnish private dentists [28]. Interestingly, we found that GDs and dental students performed nsRCTs on molar teeth in approximately half of the cases (49.8%). This is rather surprising, as in some other countries GDs do not perform nsRCTs on molars as often as endodontists [23], which in our dataset may partly reflect the high proportion of maxillary anterior teeth treated by endodontists due to trauma. Overally, most of the nsRCTs in our study were performed by GDs in purchased service whose role in the dental care has grown in the last 15 years in Finland.

The strength of the present study is that the data came from a large practice-based database of Helsinki PDS. As public services are important part of Finland’s dental care, this study provides good insights into nsRCTs among the adult population. The study design presents a reliable overview of clinical practice from almost two decades. Nevertheless, the new healthcare reform introduced in 2023 in Finland, may change how dental care is assessed in Finland. For instance, this new reform could modestly affect the number of annual nsRCTs or the operators who provide endodontic care. The register-based study design is also a weakness of this study, as all results are based on registers recorded by dental operators in the electronic patient files. Thus, any data missing or not recorded were handled as is.

The observed association between age groups and specific tooth types treated emphasize the need for age-sensitive approaches in treatment planning. The use of comprehensive register-based data demonstrates the value of longitudinal public-health records to guide evidence-based decision-making. However, limitations such as missing clinical and radiographic data or socioeconomic variables suggest that future studies should aim to integrate broader datasets to provide a more holistic view of patient and treatment profiles.

## Conclusion

A large proportion of nsRCTs were carried out at Helsinki PDS in a 16-year observation period. The prevalence of nsRCTs remained mainly unchanged. Special attention should be paid in uncompleted nsRCTs as they constitute a significant load for efficiency of PDS and a potential threat for patients health. Patient-, tooth- and operator-related factors associated with nsRCTs, together with the subgroup differences revealed in this study, should be considered when distributing clinical tasks. Thus, the study findings provide clinicians and decision-makers with evidence to support treatment planning and service management aimed at improving clinical outcomes and service efficiency.

## Data Availability

Data for this study are available from the City of Helsinki, but restrictions apply to the availability of these data, which were used under licence for the current study and so are not publicly available. The data are, however, available upon request and with the permission of the City of Helsinki.

## Appendix A

According to the Finnish National Institue for Health and Welfare (THL), the procedures are time-based and tooth spesific.

- SGA: nsRCT initiation; cleaning and shaping of root canals of the tooth. Includes possible intra-canal medication and temporary restoration.
- SGB: root canal filling
- Treatment codes: nsRCT initiation (SGA01 (emergency), SGA02 (single-canal, maximum duration of 20 minutes), SGA03 (two root canals, minimum duration of 20 minutes), SGA04 (three or more root canals, minimum duration of 30 minutes), SGA05 (three or more root canals, complicating procedure, minimum duration of 45 minutes)), root canal filling (SGB10 (one root canal), SGB20 (two root canals), SGB30 (three or more root canals))
